# Preparation and Characterization of a New Low Refractive Index Ferrofluid

**DOI:** 10.3390/ma12101658

**Published:** 2019-05-22

**Authors:** Qianhui Cao, Zhili Zhang, Jun Yu, Hongchao Cui, Xinzhi He, Decai Li

**Affiliations:** 1 School of Mechanical, Electronic and Control Engineering, Beijng Jiaotong University, Beijing 100044, China; 17121348@bjtu.edu.cn (Q.C.); 16116356@bjtu.edu.cn (J.Y.); hccui@bjtu.edu.cn (H.C.); xzhe@bjtu.edu.cn (X.H.); 2 Key Laboratory of Vehicle Advanced Manufacturing, Measuring and Control Technology, Beijing Jiaotong University, Ministry of Education, Beijing 100044, China; 3 Department of Mechanical Engineering, Tsinghua University, Beijing 100084, China; lidecai@mail.tsinghua.edu.cn

**Keywords:** ferrofluid, magnetic nanoparticles, silver, refractive index, optical fiber sensor

## Abstract

In this research, a new low refractive index ferrofluid is proposed by coating magnetic nanoparticles with a layer of silver, applying the method of modified chemical co-precipitation. This preparation method is green and environmentally friendly without toxic gases being released. Coated nanoparticles are characterized by X-ray diffraction (XRD), transmission electron microscopy (TEM), energy dispersive X-ray spectrometry (EDS), X-ray photoelectron spectroscopy (XPS), and vibration sample magnetometery (VSM). These characterizations show that the silver nanoparticles grow on the surface of magnetic nanoparticles in this new ferrofluid. The hysteresis loop of this new ferrofluid demonstrates that it maintains superparamagnetic properties. A new method of refractive index measurement is applied in this research by employing a long-period grating (LPG) optical fiber sensor. The change value in the refractive index per unit concentration reduces by 16.46% compared to the conventional ferrofluid.

## 1. Introduction

Ferrofluid is a kind of smart materials formed by magnetic nanoparticles with surfactants coated on the surface dispersing in a carrier liquid [1,2]. As a functional material, ferrofluid has aroused considerable interest for its unique properties which have been widely used in many fields, such as biomedicine [3], catalyst [4], magnetic resonance imaging [5,6], gene detection [7], cell sorter [8,9], and so on. In addition, ferrofluid exhibits remarkable magneto-optical properties. When there is no external magnetic field, the magnetic nanoparticles in the ferrofluid are uniformly distributed and the optical properties are isotropic. Yet, when there is an external magnetic field, magnetic nanoparticles are oriented in the direction of the external magnetic field and the optical properties are anisotropic [10]. However, the difference in refractive index between the ferrofluid and the air causes Fresnel reflection at the interface and reduces the transmittance of light. Low refractive index ferrofluid can effectively avoid Fresnel reflection and has promising applications in fabricating optical switches [11,12], tunable filters [13], and other optical devices [11]. Therefore, preparation of low refractive index ferrofluid is important for its application in the optical field. Researchers have predicted that ferrofluid, which contains Fe_3_O_4_ magnetic nanoparticles with a spherical isotropic metallic shell could have the property of low refractive index [14]. However, coating Fe_3_O_4_ magnetic nanoparticles with a metallic shell is difficult to realize, especially for gold or silver, due to the stability of noble metals. Despite these difficulties, the objective of this research is to propose preparation of a new low refractive index ferrofluid through a silver coating since it has an outstanding nonlinear optical property. In this research, a modified method of co-precipitation is applied to synthesize this new ferrofluid by reducing AgNO_3_ solution using glucose. 

In addition, a new method employing a long-period grating (LPG) optical fiber sensor is adopted to measure the refractive index of ferrofluid. Previously, a method of employing a reflection technique was reported to measure the refractive index of ferrofluid. In 2002, Yang successfully measured the refractive index of ferrofluid by total reflection technique [15]. However, the method requires sophisticated instrumentation, elaborate optical alignment and complicated data processing [16]. In contrast, this method employing a LPG optical fiber sensor is easy to operate and guarantees the accuracy of results. 

## 2. Materials and Experiments

### 2.1. Materials

Ferric chloride hexahydrate (FeCl_3_·6H_2_O), ferrous chloride tetrahydrate (FeCl_2_·4H_2_O), silver nitrate (AgNO_3_), ammonia solution and glucose were purchased from Beijing Chemical Reagents Company (Beijing, China). Polyethylene glycol (PEG, powder, average *M_w_*=4000) were purchased from Shanghai Macklin Biochemical Technology Company (Shanghai, China). All chemical reagents used in this research were analytical reagent grade without further purification. Ultrapure water was used throughout the whole experiment. 

### 2.2. Experiments

**Figure 1:**
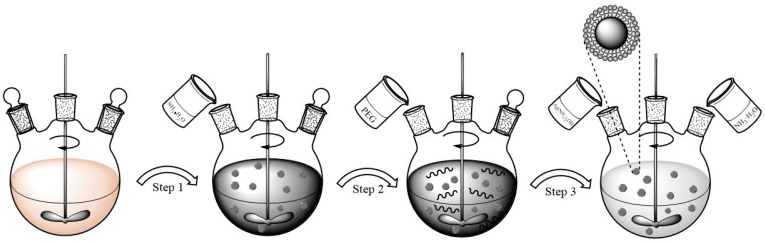
Schematic synthesis of Fe_3_O_4_@Ag nanoparticles.

The most common method to prepare ferrofluid is chemical co-precipitation. In this method, a mixture of salts suspended in an aqueous alkaline medium is prepared. Subsequently, different procedures such as decantation, magnetic separation, centrifugation, and dilution are applied to the suspension [17,18]. In this research, a modified method of co-precipitation was adopted to synthesize this new low refractive index ferrofluid by reducing AgNO_3_ solution using glucose. Figure 1 shows the schematic synthesis of Fe_3_O_4_@Ag nanoparticles. Ferric chloride hexahydrate and ferrous chloride tetrahydrate were mixed in ultrapure water with a molar ratio of 1:1.6 for adequate reaction. After these two solid reagents were dissolved completely, 25% ammonia solution used as the precipitant was added to the mixture solution with vigorous stirring. After that, PEG was added to the solution under vigorous stirring for about 40 minutes. Then, 25% ammonia solution was added with a dropper to 100 mL 0.1 mol/L AgNO_3_ aqueous solution. After stirring, the mixed silver ammonia solution and glucose were added to the mixture solution. The mixture solution was heated to 50 °C and the color of the solution slowly changed from black to dark grey. The mixture was stirred slightly for 0.5 h to obtain the appropriate thickness of coating. Finally, Fe_3_O_4_@Ag nanoparticles were separated with an external magnet and washed by either ultrapure water or ethanol several times. Fe_3_O_4_@Ag nanoparticles and the carrier liquid were fully ground in certain ratios with high-energy ball milling for about 4 hours.

### 2.3. Characterization

The morphology was characterized with a JEM-2100 Transmission Electron Micrograph (TEM, JEOL, Tokyo, Japan) and with energy dispersive X-ray spectroscopy (EDS, JEOL, Tokyo, Japan). X-ray diffraction (XRD) measurement was performed by a D8 Advance Bruker AXS diffractometer (Rigaku, Tokyo, Japan) at 40 kV, 100 mA using a Cu-target tube and a graphite monochromator (Rigaku, Tokyo, Japan). Scans were made in the 2θ range of 20–80° with a step size of 0.2° and a count time of 2 second per step. The qualitative analysis of the XRD patterns was performed based on the PDF-2 reference database from the International Center for Diffraction Data database. X-ray photoelectron spectroscopy (XPS) analysis was conducted using a PHI Quantera SXM multi-technique system with an Mg Ka X-ray source (Perkin-Elmer Physical Electronics, ULVAC-PHI, Kanagawa, Japan) to investigate the chemical properties of Fe_3_O_4_@Ag nanoparticles. Magnetic properties were measured by a vibration sample magnetometer (VSM, Lakeshore 7307, Lakeshore Cryotronics, Westerville, America) at room temperature. The ASE broadband light source (KOHERAS, SuperK Uersa, NKT Photonics, Birkerod, Denmark) and the spectrometer (OSA, YOKOGAWA AQ6375, Yokogawa, Tokyo, Japan) were employed in the refractive index measurement process.

## 3. Results and Discussion 

### 3.1. Reaction Mechanism of the Preparation Process

After ammonia solution is added, its reaction with Fe^3+^ and Fe^2+^ forms Fe_3_O_4_. With high surface energy, the Fe_3_O_4_ aggregate rapidly, and thus form particles like seeds. PEG attached to the Fe_3_O_4_ particles surfaces could prevent their further growth. When PEG is dissolved in aqueous solution, it is easy to form strong hydrogen bonds on the surface of Fe_3_O_4_ particles. The hydrogen bonds can produce a protective film of polymer which surrounds Fe_3_O_4_ particles, at the same time the molecular bonds can spread into the aqueous solution which makes Fe_3_O_4_ particles monodisperse because of the steric hindrance effect. AgNO_3_ also reacts with ammonia solution to form AgOH. Since AgOH is very unstable, it can further react with ammonia solution to produce Ag(NH_3_)_2_OH. Then Ag ions are reducted in situ on the surface of Fe_3_O_4_ particles, resulting from their strong binding interaction with added glucose. 

### 3.2. X-ray Diffraction (XRD) Analysis

**Figure 2:**
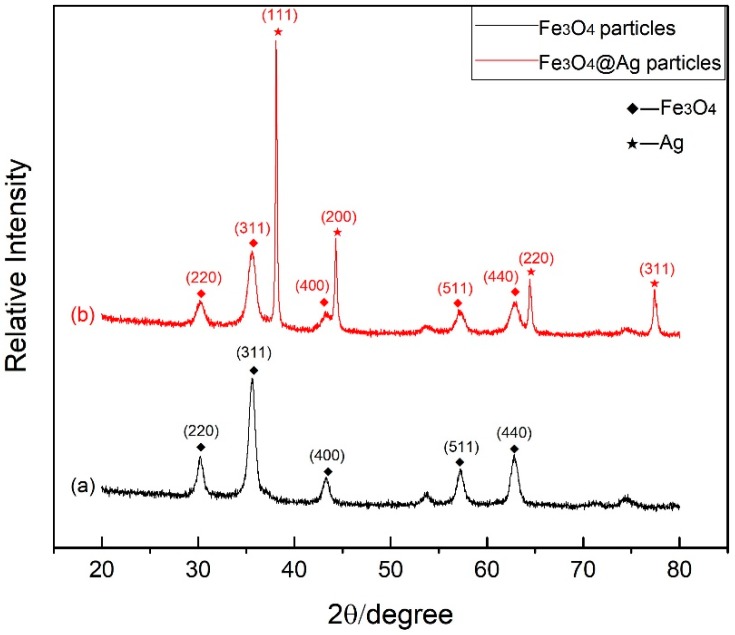
XRD patterns of (**a**) Fe_3_O_4_ particles and (**b**) Fe_3_O_4_@Ag particles.

Both Fe_3_O_4_ nanoparticles and Fe_3_O_4_@Ag nanoparticles are investigated by XRD patterns to explain the crystalline nature. XRD patterns of Fe_3_O_4_ nanoparticles and Fe_3_O_4_@Ag nanoparticles are shown in Figure 2. As indicated in Figure 2a, peaks at 2θ=30.1°, 35.5°, 43.1°, 56.9° and 62.6° can be indexed to (220), (311), (400), (511) and (440) crystalline planes, respectively, which are in good agreement with the face-centered cubic structure of Fe_3_O_4_ (JCPDS Card No. 19-0629). As indicated in Figure 2b, peaks at 2θ values of 38.2°, 44.6°, 64.5° and 77.5° can be defined as the Ag characteristic peaks corresponding to the reflections of the (111), (200), (220) and (311) crystalline planes of Ag (JCPDS Card No. 65-2871). The PEG layer has no effect on the crystal structure of Fe_3_O_4_@Ag nanoparticles because PEG is a kind of amorphous polymer material. The intensity of Fe_3_O_4_ in the Fe_3_O_4_@Ag particles decreases, possibly because of the silver covering on the particles.

### 3.3. Transmission Electron Microscopy (TEM) and Energy Dispersive X-Ray Spectrometry (EDS) Images

**Figure 3:**
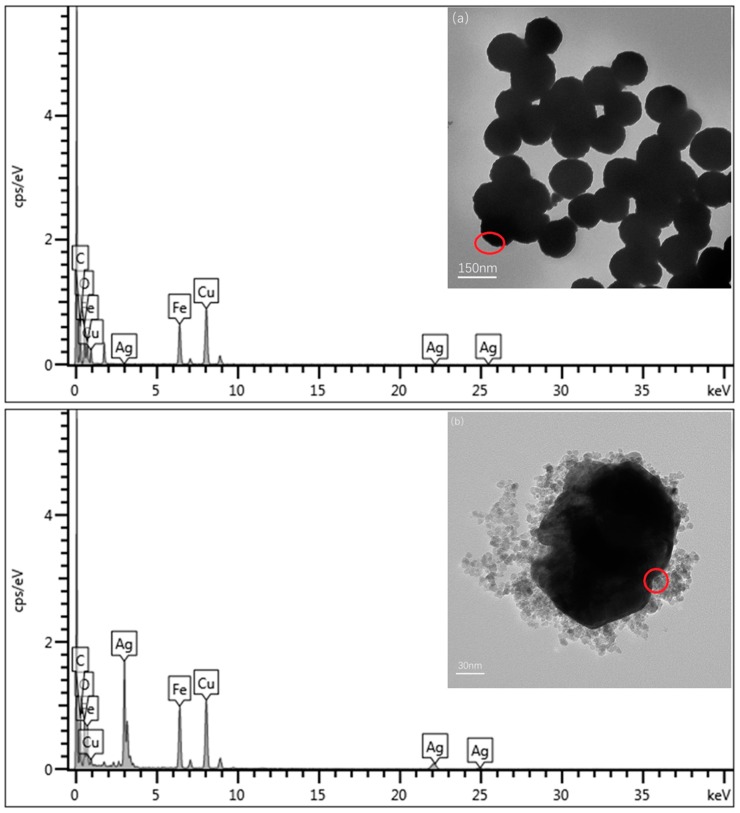
TEM and energy dispersive X-ray spectrometry (EDS) images of (**a**) Fe_3_O_4_ particles and (**b**) Fe_3_O_4_@Ag particles deposited on carbon-coated Cu grids.

Morphology of Fe_3_O_4_ particles and Fe_3_O_4_@Ag particles is characterized by TEM operated with a microanalytic system EDS Link ISIS EDX, at a voltage of 200 kV. The particles are dispersed in ultrapure water with ultrasonic treatment for 30 minutes. Then, a drop of the colloidal suspension is dripped on a carbon-coated Cu grid and allowed to dry before observation. Figure 3a is the image of Fe_3_O_4_ nanoparticles before reducing AgNO_3_ solution, while Figure 3b is the image of Fe_3_O_4_@Ag nanoparticles after reducing AgNO_3_ solution. Observation reveals that the surface of Fe_3_O_4_@Ag particles are not smooth, which demonstrates that the silver shell is composed of many individual particles. EDS analysis in Figure 3a,b is the elemental composition of the edge of the particles, shown in the red areas in the images. In the EDS spectrum, the Fe and O peaks indicate the Fe_3_O_4_ magnetic nanoparticles, and the existence of C and Cu peaks is due to the carbon-coated Cu grid. More importantly, the observed Ag peak demonstrates the composition of the Ag element which illustrates that the Fe_3_O_4_ magnetic particles are coated by a layer of Ag nanoparticles. Element contents of Fe_3_O_4_ particles and Fe_3_O_4_@Ag particles analyzed by EDS are also shown in Table 1. 

### 3.4. X-ray Photoelectron Spectroscopy (XPS)

**Figure 4:**
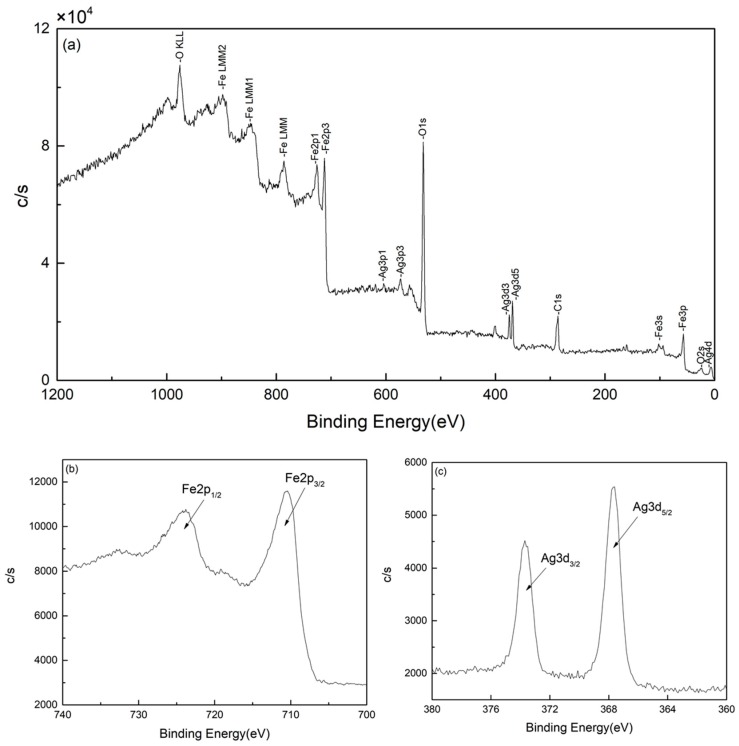
XPS spectra of (**a**) Fe_3_O_4_@Ag particles (**b**) Fe 2p peaks of Fe_3_O_4_@Ag particles and (**c**) Ag 3d peaks of Fe_3_O_4_@Ag particles.

In order to achieve a better understanding of the chemical state of Fe_3_O_4_@Ag particles, XPS analysis was performed. Figure 4a shows the XPS spectra of Fe_3_O_4_@Ag nanoparticles. It can be observed that after coating with Ag, the intensity was lower than the original Fe_3_O_4_ [19]. C1_S_ and O1_S_ peaks can be observed due to the addition of PEG. The Fe 2p and Ag 3d in Fe_3_O_4_@Ag nanoparticles are compared in Figure 4b and Figure 4c. The binding energies at 724.4 eV and 710.6 eV in Figure 4b are attributed to Fe 2p_1/2_ and Fe 2p_3/2_ which are the characteristics of Fe_3_O_4_. No obvious difference can be found for Fe 2p after Ag coating. A change in intensity and broadening in satellite peak near 718.8 eV is attributed to the overlap of Fe 2p3/2 and Ag 3s peaks [19]. Figure 4c displays the spectra of Ag 3d for Fe_3_O_4_@Ag nanoparticles. The Ag 3d binding energy region consists of an asymmetric broad peak centered around 373.9 eV and 367.9 eV for Ag 3d_3/2_ and 3d_5/2,_ respectively. The peaks of Ag 3d_5/2_ and Ag 3d_3/2_ indicate that Ag is the main component of the surface. These peaks can be split to about ~6 eV that shows the metallic condition of silver in the form of Ag in the present sample [20,21].

### 3.5. Magnetic Properties

**Figure 5:**
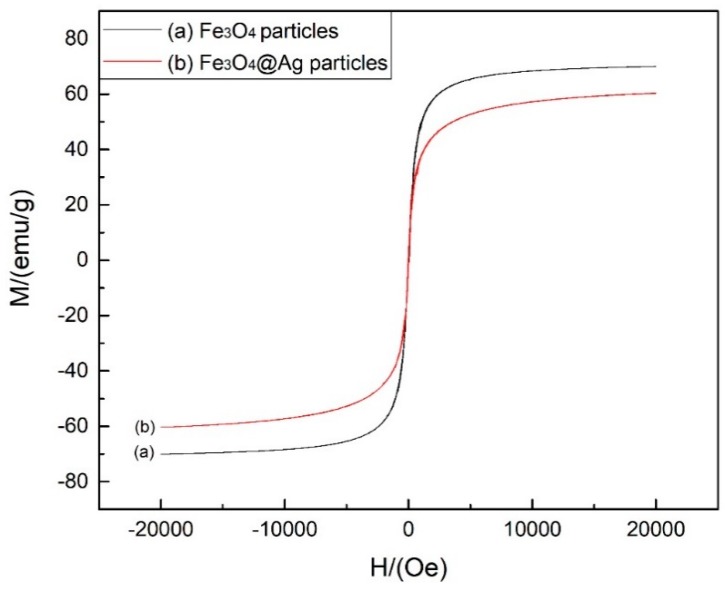
Hysteresis loops of (**a**) Fe_3_O_4_ particles and (**b**) Fe_3_O_4_@Ag particles.

Hysteresis loops of Fe_3_O_4_ particles and Fe_3_O_4_@Ag particles measured by VSM at 300 K are shown in Figure 5. Saturation magnetization (Ms) of Fe_3_O_4_@Ag particles reduces slightly. They have no remnant magnetism properties that can be used in the sensors of magnetic field. The reduction in Ms results from the decrease in magnetic particle density. The energy of magnetic materials in an external magnetic field is proportional to the number of magnetic molecules in a single magnetic domain. This decrease reflects a smaller percentage of net magnetic material per gram of overall sample. The large surface-to-volume ratio of Fe_3_O_4_@Ag particles is possibly another factor that leads to the decrease in Ms.

### 3.6. Refractive Index Measurements

**Figure 6:**
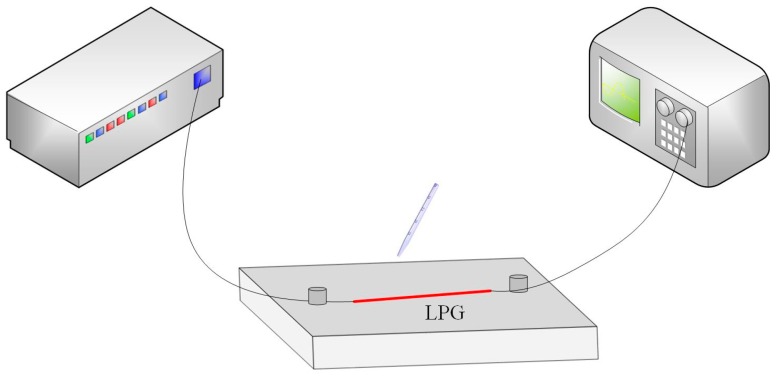
Schematic diagram of refractive index measurements.

**Figure 7:**
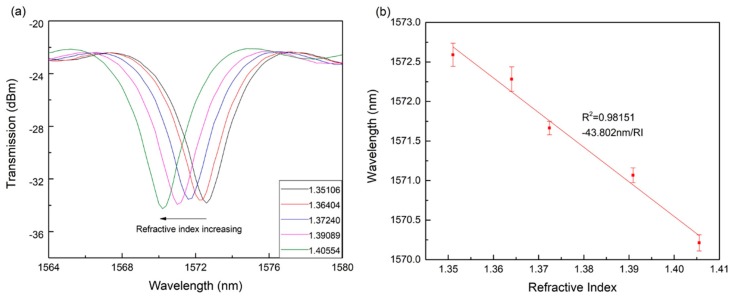
The transmission spectrum and the fitting curve of glycerol aqueous solutions. (**a**) Transmission spectrum of the long-period grating (LPG) optical fiber sensor in different refractive index glycerol aqueous solutions. (**b**) Fitting curve of the wavelength shift of peak and the change value of refractive index.

The LPG optical fiber sensor consists of an LPG fused between two single mode fibers as shown in Figure 6. The two ends of the single mode fibers are connected with the broadband light source and the spectrometer. The broadband source (KOHERAS, SuperK) is an ultra-continuous spectral white light source with an ultra-wideband output spectrum. It has an output connector with a fiber optic connector for easy interfacing. The spectrometer (YOKOGAWA AQ6375, Yokogawa, Tokyo, Japan) is a long-wavelength benchtop spectrum analyzer with wavelength ranging from 1200 nm to 2400 nm. These optical fibers are placed in the glass plate without strain. The relationship between refractive index and wavelength is studied by using different concentrations of glycerol aqueous. Then the refractive index of two ferrofluids is obtained from the fitting curve shown in Figure 7. After each experiment, the LPG optical fiber is washed with ultrapure water and dried with an ear washer. The transmission spectrum is restored to the initial position to guarantee that no residual glycerol solution is present on the surface of LPG, and then the following experiment is carried out.

Five different concentrations of glycerol aqueous solutions are injected in the plate by a dropper and the LPG is completely immersed. The concentrations of glycerol are 15%, 25%, 35%, 45% and 55%. The corresponding refractive index is 1.35106, 1.36404, 1.37240, 1.39089 and 1.40554. When the external refractive index changes, the transmission spectrum of the LPG optical fiber sensor also drifts. Figure 7a illustrates the transmission spectrum of the LPG optical fiber sensor in different refractive index glycerol aqueous solutions. It can be observed that with the increase of refractive index, the transmission spectrum drifts to the short wavelength direction. The effective refractive index of the dominant cladding mode increases with the increase of the external refractive index. The effective refractive index of the core mode depends on the refractive index of the core and cladding of the fiber and is not affected only by the change of the external refractive index. When the external refractive index increases, the refractive index difference between the dominant cladding mode and the core mode decreases and the loss peak drifts to the short wavelength. Figure 7b shows the fitting curve of the wavelength shift of peak and the change of refractive index. The standard deviation is 0.1189, 0.1549, 0.0849, 0.0931 and 0.1029. In addition, it indicates the significant linear correlation between the refractive index and the wavelength.

**Figure 8:**
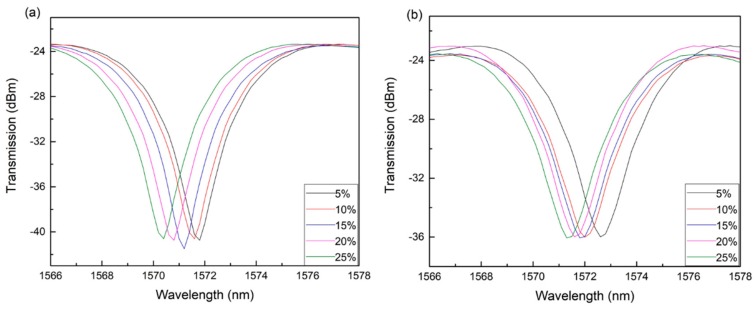
Transmission spectrum of the LPG optical fiber sensor in (**a**) ferrofluid and (**b**) Fe_3_O_4_@Ag ferrofluid.

**Figure 9:**
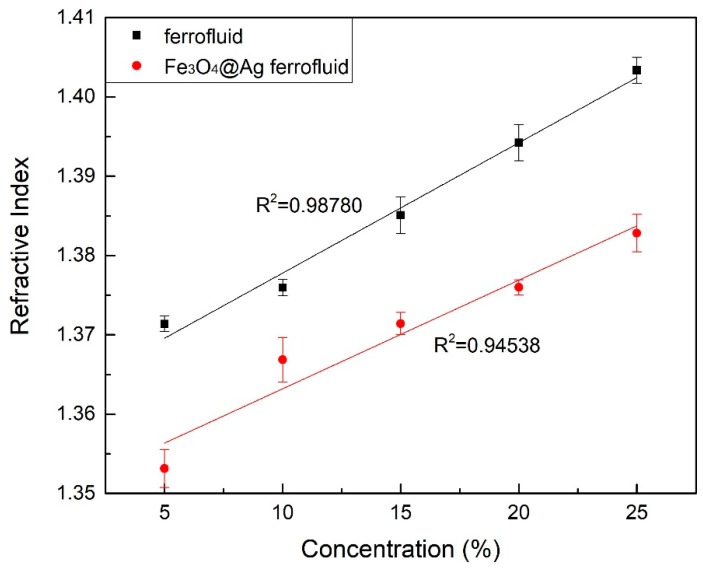
Fitting curves of the refractive index of these two ferrofluids.

Refractive index of five different concentrations of the conventional ferrofluid solutions and the new ferrofluid solutions are measured in the same process. The concentrations of these two ferrofluid are 5%, 10%, 15%, 20% and 25%. The refractive index of these two ferrofluids can be obtained by measuring the output wavelength according to the fitting curve shown in Figure 7b. Figure 8 reveals the transmission spectrum of the LPG optical fiber sensor in different concentration solutions of the conventional ferrofluid and the new ferrofluid. The transmission spectrum of the sensor drifts because of the change of the refractive index to be measured. As indicated in Figure 8a, the wavelength value of transmission spectrum peak of the LPG optical fiber sensor is 1572.6 nm, 1572.0 nm, 1571.8 nm, 1571.6 nm and 1571.3 nm when the concentrations of the conventional ferrofluid are 5%, 10%, 15%, 20% and 25%. As shown in Figure 8b, the wavelength value drops to 1571.8 nm, 1571.6 nm, 1571.2 nm, 1570.8 nm and 1570.4 nm in the new ferrofluid with the same concentration of 5%, 10%, 15%, 20% and 25%. In the conventional ferrofluid, when the wavelength drift is 1.3 nm, the refractive index changes from 1.371 to 1.403 while in the new ferrofluid with the same concentration, when the wavelength drift is 1.4 nm, the refractive index changes from 1.353 to 1.383. Table 2 reveals the refractive index of these two ferrofluids. The change value in refractive index per unit concentration reduces by 16.46% compared to the conventional ferrofluid. Figure 9 illustrates fitting curves of the refractive index of these two ferrofluids. The standard deviations of the conventional ferrofluid are 0.0009, 0.0010, 0.0023, 0.0022 and 0.0016 while the standard deviations of the new ferrofluid are 0.0023, 0.0028, 0.0014, 0.0009 and 0.0024. Meanwhile, these fitting curves reveal the significant linear correlation with the linear fitting of 0.98780 and 0.94538 in the conventional ferrofluid and the new ferrofluid respectively. 

The transmission spectrum of the LPG fiber is generally the superposition of multiple mode interference spectrums. The interference intensity among modes can be expresses as:

(1) $$I=\sum_{i=1}^{n}I_{i}+2\sum_{i=1}^{n-1}\sum_{j=i+1}^{n}\sqrt{I_{i}I_{j}}\cos[2\pi (n_{i}-n_{j})L/\lambda ],$$

where $I_{i}$ and $I_{j}$ are the light intensity of the i and j modes, $n_{i}$ and $n_{j}$ are the effective refractive index of the i and j modes. L is the interference length and λ is the wavelength of the transmitting light. Thus, the light intensity of the LPG optical fiber can be simplified as [22]:

(2) $$I=I_{1}+I_{2}+2\sqrt{I_{1}I_{2}}\cos(\phi ),$$

where $I_{1}$ and $I_{2}$ are light intensities of two modes. ϕ is the phase difference between the two modes which can be expressed as [23]:

(3) $$\phi =\frac{2\pi \Delta n_{eff}L}{\lambda },$$

where $\Delta n_{eff}$ is the effective refractive index difference of two main modes participating in the interference. When the phase difference between the two modes satisfies ϕ=(2m+1)π, the loss peak appears. The wavelength of the loss peak can be expressed as:

(4) $$\lambda_{m}=\frac{2\Delta n_{eff}L}{2m+1}.$$

When the external refractive index changes, the effective refractive index of LPG optical fiber modes will also change. Since the silver shell can provide a low refractive index, the effective refractive index will increase resulting in the increase of the wavelength drift of this new ferrofluid.

## 4. Conclusions

In this research, the design of a new low refractive index ferrofluid is proposed by coating Fe_3_O_4_ magnetic nanoparticles with a silver shell. Despite the difficulty of coating magnetic nanoparticles with a noble metal shell, this new ferrofluid is successfully prepared by a modified method of chemical co-precipitation and reduction. The Ms of this new ferrofluid reduces slightly from 70.03 emu/g to 58.90 emu/g. The hysteresis loop of the new ferrofluid also reveals that there are no remnant magnetism and the samples still have the superparamagnetic property. A new method employing an LPG optical fiber sensor is firstly adopted to measure the refractive index of ferrofluid. The significant linear correlation between the refractive index and the wavelength is obtained in the experiment of glycerol aqueous solutions. The refractive index of both the conventional ferrofluid and the new ferrofluid is obtained according to the fitting curve with the same process. Compared to the previous methods, this method is easy to operate, and guarantees accuracy of the results. The results of refractive index experiments illustrate that the refractive index of ferrofluid with Fe_3_O_4_@Ag nanoparticles decreases by nearly 0.02 with the same concentration, and the change value of refractive index per unit concentration reduces by 16.46%.

## Figures and Tables

**Table 1:** Elements contents of Fe_3_O_4_ nanoparticles and Fe_3_O_4_@Ag nanoparticles in the red areas.

Samples	Elements Contents (wt.%)				
	Fe	Ag	O	Cu	C
Fe_3_O_4_	22.79	0	17.00	38.01	22.20
Fe_3_O_4_@Ag	17.71	33.77	10.77	23.65	14.11

**Table 2:** Refractive index of ferrofluid and Fe_3_O_4_@Ag ferrofluid.

Samples	Concentration (%)	Refractive Index	Change in Refractive Index Per Unit Concentration
	5	1.371	
	10	1.376	
ferrofluid	15	1.385	0.00164
	20	1.394	
	25	1.403	
	5	1.353	
	10	1.367	
Fe_3_O_4_@Ag ferrofluid	15	1.371	0.00137
	20	1.376	
	25	1.383	
